# Research and Remedies

**Published:** 1912-02-24

**Authors:** 


### RESEARCH AND REMEDIES.

### Acute Nephritis and its Ultimate Results.

ACUTE nephritis, or acute Bright's disease, may supervene on a long-standing chronic case, or may appear *de novo* in a previously healthy person. When the latter happens the patient is nearly always young, usually under twenty years of age and nearly always under thirty. The disease is a serious one, and not a few patients die, either during the acute attack or of some relapse or complication before a complete return to health has taken place. The exact prospects of those (the majority) who do recover have long been matters of discussion. Some physicians hold that most of them eventually—perhaps many years later—develop chronic Bright's disease and ultimately die of it; others deny that this miserable outlook really confronts those who have recovered from acute nephritis. The question is clearly a statistical one, but the difficulty is to follow up enough cases for conclusions and averages to be formulated. Ernberg has lately undertaken such an investigation, and is on the side of those who take a cheerful view. He concludes that acute nephritis in youth kills either at once or after a long illness during which the patient cannot properly be described at any time as healthy; or else that it passes completely away leaving no permanent damage to the kidneys in most cases. He traced for twenty years 106 patients under fifteen years of age; seventeen died of the original nephritis, and of the other 89 he was able to find and examine 61. Of these 43 were in perfect health and their urinary systems seemed to be quite normal. Out of 50 similar cases in patients between fifteen and thirty years of age, 12 died of the original attack; and once more he found that the majority of the survivors whom he was able to trace had grown up quite healthy.

### Gloves in Obstetric Practice.

IT is familiar teaching now that a boiled glove is an absolute necessity for the obstetrician, and that no vaginal examination of a parturient woman should be made without one. The exact rationale of this proceeding is strongly questioned by Dr. D. H. Stewart, of New York, who contributes an account of some ingenious experiments to the *American Journal of Obstetrics and Gynæcology.* This observer felt that there was a serious flaw in the argument which alleges that the vulva is septic and the vagina sterile, and that therefore only an aseptic finger (that is, a gloved one) should be used for examination. To test his belief that such a gloved finger cannot possibly remain sterile as it passes through the vulva, he conducted a large number of culture tests. He soon satisfied himself that the normal vulva is germ-contaminated (though not always, he finds, by pathogenic organisms), and that the normal vagina is sterile. But he also found that even with every possible precaution, a gloved finger passed through the vulva into the vagina becomes immediately contaminated itself and also contaminates the vagina. He also showed that in an ordinary room a boiled glove becomes contaminated from the air in from two to thirty minutes and cannot be relied on as aseptic five minutes after it is put on.

### The Alternative to Boiled Gloves.

DR. STEWART then proceeds to develop his argument that antisepsis is preferable to asepsis for obstetric practice. He is not entirely adverse to the wearing of gloves, though he thinks it quite possible to do without them: indeed the protection of the physician from infection is their principal recommendation in his view. He holds that the finger, or the glove, of the obstetrician should be thoroughly plastered with some strong antiseptic: chlorinated lime is the bactericide he prefers. A paste of this substance he has smeared thoroughly all over his finger, and has maintained the finger sterile to culture media for twelve hours at a time. He finds that daily use of this drastic germicide makes the skin sore, but that in occasional use it does not possess this disadvantage. By experiments he proves that vaginal examination with a finger thus protected does not contaminate that passage with bacteria, nor does it prove uncomfortable to the patient. As a matter of practice, the plain boiled glove is not much used in this country; a lysol solution is nearly always used as an additional precaution. It would be of great interest if this author or some other would carry out a similar research into the merits or defects of this antiseptic as ordinarily used in obstetric cases.

### An Ophthalmo-Reaction for Typhoid Fever.

THREE or four years ago the Calmette ophthalmo-reaction for tuberculosis was the topic of the moment with the medical profession; and scores of physicians were investigating for themselves the value claimed for this test. To-day it has been practically abandoned as distinctly dangerous, and not by any means infallible. Yet the principle upon which it was founded remains, and Dr. Austrian publishes in the *Johns Hopkins Hospital Bulletin* his investigations into an ophthalmo-reaction designed on similar lines for the detection of typhoid fever. His matured judgment is to the following effect: A solution of one-third to one-half of a milligram of "typho-protein" in a drop of water, when instilled into the conjunctival sac of a patient ill with typhoid fever, causes a typical inflammatory reaction. The most constant results are obtained when the "protein" is derived from numerous different strains of typhoid bacilli. The typical response shows definite characteristics: thus it is limited to or is maximal in the palpebral conjunctiva of the lower lid and in the caruncle. It appears in one to five hours, reaches its maximum intensity in about six hours, and persists twenty-four hours or longer. The most characteristic sign is the deep purple congestion of the palpebral conjunctiva of the lower lid and of the caruncle. The reaction is relatively specific and can be differentiated from that produced in other diseases and in health.

